# Increased CSF osmolarity reversibly induces hydrocephalus in the normal rat brain

**DOI:** 10.1186/2045-8118-9-13

**Published:** 2012-07-11

**Authors:** Satish Krishnamurthy, Jie Li, Lonni Schultz, Kenneth A Jenrow

**Affiliations:** 1 Department of Neurosurgery, Upstate Medical University, Syracuse, NY, 13210, USA; 2 Department of Biostatistics and Research Epidemiology, Henry Ford Hospital, 2799, West Grand Blvd, Detroit, MI, 48202, USA; 3 Department of Neurosurgery, Henry Ford Hospital, 2799, West Grand Blvd, Detroit, MI, 48202, USA

**Keywords:** Hydrocephalus, Brain, CSF, Osmotic gradient, Ventricular volume, Rat

## Abstract

**Background:**

Hydrocephalus is a central nervous system (CNS) disorder characterized by the abnormal accumulation of cerebrospinal fluid (CSF) in cerebral ventricles, resulting in their dilatation and associated brain tissue injury. The pathogenesis of hydrocephalus remains unclear; however, recent reports suggest the possible involvement of abnormal osmotic gradients. Here we explore the kinetics associated with manipulating CSF osmolarity on ventricle volume (VV) in the normal rat brain.

**Methods:**

CSF was made hyper-osmotic by introducing 10KD dextran into the lateral ventricle, either by acute injection at different concentrations or by chronic infusion at a single concentration. The induction and withdrawal kinetics of dextran infusion on VV were explored in both contexts.

**Results:**

Acute intraventricular injection of dextran caused a rapid increase in VV which completely reversed within 24 hours. These kinetics are seemingly independent of CSF osmolarity across a range spanning an order of magnitude; however, the magnitude of the transient increase in VV was proportional to CSF osmolarity. By contrast, continuous intraventricular infusion of dextran at a relatively low concentration caused a more gradual increase in VV which was very slow to reverse when infusion was suspended after five days.

**Conclusion:**

We conclude that hyperosmolar CSF is sufficient to produce a proportional degree of hydrocephalus in the normal rat brain, and that this phenomenon exhibits hysteresis if CSF hyperosmolarity is persistent. Thus pathologically-induced increases in CSF osmolarity may be similarly associated with certain forms of clinical hydrocephalus. An improved understanding of this phenomenon and its kinetics may facilitate the development of novel therapies for the treatment of clinical hydrocephalus.

## Introduction

Hydrocephalus is a CNS disorder characterized by the abnormal accumulation of cerebrospinal fluid (CSF) in cerebral ventricles and increased ventricle volume (VV). The pathogenesis underlying the emergence of hydrocephalus is poorly understood [1-5] and remains an unresolved issue despite many years of investigation [6-10]. Clinical hydrocephalus is commonly classified based on symptomatology as either obstructive hydrocephalus, communicating hydrocephalus, or normal pressure hydrocephalus (NPH). Classically, hydrocephalus is thought to reflect either a blockage of CSF circulation within the ventricles and/or impaired CSF absorption through the arachnoid projections into the cranial venous sinuses or nasal lymphatics [11-19]. However, clinical hydrocephalus routinely manifests without any detectable blockage of CSF circulation pathways [20-24], and several reports have cast doubt on the role of arachnoid projections in this context [25-27].

The classical view regarding the etiology of hydrocephalus is further challenged by recent reports that chronically increased CSF osmolarity is sufficient to produce hydrocephalus in the normal rat or dog brain [28-31]. It has been proposed that hydrocephalus in this context reflects a disparity between the relatively free movement of water within brain tissues (including the ventricles), made possible by the presence of aquaporins, and the strictly regulated movement of hydrophilic macromolecules across the blood–brain barrier (BBB) separating CSF and plasma space. As a result of this disparity, artificially increasing CSF osmolarity by infusing hydrophilic macromolecules creates an osmotic gradient which draws water into the ventricles.

Here we investigate the kinetics of hydrocephalus following the injection/infusion of 10KD dextran into the lateral ventricle in the normal rat brain. Our results lend additional support to the hypothesis that osmotic effects are sufficient to induce hydrocephalus and reveal that the associated kinetics can have a dramatic effect on the nature and persistence of the hydrocephalic response.

## Materials and methods

### Subjects

Adult female Sprague–Dawley rats (220-250 g, n = 84; Harlan, Indianapolis, Indiana, USA) were used in all experiments. Rats were housed in an AAALAC-accredited animal care facility at Wayne State University and maintained on a 12-h light/dark cycle with food and water provided *ad libitum*. Animal procedures were carried out in accordance with NIH guidelines for the care and use of experimental animals and the experimental protocol was approved by the Institutional Animal Care and Use Committee at Wayne State University in Detroit, Michigan.

### Experiment 1: Acute intraventricular injection (n = 45)

For this experiment, rats were randomly divided into five groups which received acute injections (15μl) of FITC-labeled dextran (10KD) into the lateral ventricle at different osmolarities via a Hamilton syringe. Group I (337 mOsm/L, n = 10), Group II (628 mOsm/L, n = 10), Group III (977 mOsm/L, n = 9), Group IV (2000 mOsm/L, n = 8) and Group V (3347 mOsm/L, n = 8). Ventricle volumes were assayed by volumetric analysis of MRI image data obtained from scans performed prior to injecting hyperosmotic dextran solution, at 30 minutes post-injection, and at 24 hours post-injection.

### One-time lateral ventricle injection (acute experiment)

Rats were anesthetized with Ketamine (87 mg/kg, intraperitoneally or i.p.) and Xylazine (13 mg/kg, i.p.) and mounted in an animal stereotactic instrument (Kopf). Using aseptic techniques, 15 μl of fluorescently-labeled 10KD dextran solution (1 μg/μl)15 μl of 10KD (1 μg/μl) fluorescently-labeled 10KD dextran solution was injected (1 μl/s) into the frontal horn of the right lateral ventricle using a Hamilton syringe connected to a stainless steel needle (27 gauge, blunt tip). The needle was inserted through a burr hole positioned 0.9 mm posterior, 1.2 mm lateral to bregma, and lowered perpendicular to the cranial surface to a depth of 3.6 mm ventral to the dorsal surface of the dura mater [32]. After injection, the needle was held in place for 1 minute and then slowly withdrawn. The burr hole was then sealed with bone wax and the incision was sutured closed.

### Experiment 2: Chronic intraventricular infusion (n = 39)

For this experiment, rats were randomly divided into 4 groups defined by their intraventricular infusion status (On/Off) during three consecutive 5-day intervals (A, B, C). Groups I and II: On-On-On (n = 10 for both groups); Group III: On-Off-On (n = 9); Group IV: On-Off-Off (n = 10). During ‘On’ intervals, rats received intraventricular infusion at a rate of 0.5 μl/hr with either iso-osmotic (307 mOsm/L; Group I) or hyper-osmotic (337 mOsm/L; Groups II, III, and IV) FITC-labeled dextran (10KD). During ‘Off’ intervals, intraventricular infusion of dextran was suspended with the cannula in place. Pumps were On during interval A for all groups. The infusion status was changed (Groups III and IV) at the beginning of interval B, and again (Group III) at the beginning of interval C. For each of these procedures, rats were anesthetized with ketamine (87 mg/kg, i.p.) and xylazine (13 mg/kg, i.p.) and a 1.0 cm incision was made on the dorsal surface of the neck to expose the catheter, which was either closed by ligating (‘Off’ condition) or open by unligating (‘On’ condition) the catheter using 3–0 silk suture. The efficacy and reversibility of this ligation procedure in this context was verified in preliminary experiments. Sham procedures were performed for groups where the catheter remained open during intervals A, B and C (Groups I and II). MRI scans were performed prior to pump implantation (baseline) and at the end of each interval to assay ventricular volumes.

### Osmotic pump implantation

Rats were anesthetized with ketamine (87 mg/kg, i.p.) and xylazine (13 mg/kg, i.p.) and mounted in an animal stereotactic instrument (Kopf). Using aseptic techniques, a midline incision was made and the dorsal surface of the skull was exposed completely. A blunt tip 27-gauge needle was inserted through a burr hole positioned 0.9 mm posterior, 1.2 mm lateral to bregma, and lowered perpendicular to the cranial surface to a depth of 3.6 mm ventral to the dorsal surface of the dura mater, placing the tip of the needle in the ventral horn of the lateral ventricle [32]. The needle was slowly withdrawn and replaced with a customized microcatheter of equivalent diameter connected to an Alzet osmotic minipump primed with approximately 230 μl of dextran solution (0.083 μg/μl) (Model 2002, DURECT Corporation, Cupertino, CA, USA). The microcatheter was positioned within a shallow groove created on the surface of the cranial bone, extending 3 mm posterior to the burr hole, and cemented in place using Surgicel (ETHICON, Inc., Somerville, NJ, USA) with cyanoacrylate adhesive (DURECT Corporation). The minipump was then inserted into a subcutaneous pocket in the mid-scapular region and the incision was sutured closed.

### MRI imaging and ventricular volume calculation

Whole head MRI images were acquired under Ketamine (87 mg/kg, i.p.) and Xylazine (13 mg/kg, i.p.) anesthesia in the 7-Tesla MRI System (ClinScan, Bruker, Karlsruhe, Germany) at the Magnetic Resonance Research Facility at Wayne State University. For the Experiment 1, MRI scans were performed prior to injection and at 30 minutes and 24 hours post-injection. For experiment 2, MRI scans were performed prior to cannula implantation and at 5, 10 and 15 days post-implantation, immediately prior to initiating surgical procedures to change infusion status. Coronal axial T2-weighted images were acquired using the following parameters: TR 3.53 sec, TE 38 ms, FOV 32x32mm^2^, matrix size 256x256, 1 signal average, 1 mm slice thickness, interleaved 24 slices, TA 2 m. Regions of interest (ROI) for all transverse slices were manually outlined and the surface area determined by counting the number of pixels enclosed by the ROI. Segmental volumes were calculated by multiplying slice areas by slice thickness, and total T2-weighted MRI volumes were determined by summing the segmental volumes. Internally developed MR SPIN (Signal Processing in NMR) software (MRI Institute for Biomedical Research, Detroit, MI, USA) written in Visual C++ on the Microsoft Windows platform was used for MRI and ventricular volume calculation [33]. Animals that died during the experiment were not included in the analysis.

### Statistical methods

For Experiment 1, descriptive statistics of mean, median and standard deviation were computed for each of the five treatments groups (337, 628, 977, 2000, and 3347 mOsm/L) at each of the three time-points (pre-, 30 min post-, and 24hour post-injection). Because of the wide range of variability among the groups, Wilcoxon two sample tests were performed to assess whether the pairwise group comparisons differed at each time-point. Signed rank tests were done to assess the differences over time within treatment groups. Spearman’s correlation coefficients were computed between the osmolality levels (treatment groups) and the volumes at each time point. *P*-values between 0.01 and 0.05 were interpreted as significant whereas *P*-values < 0.01 were considered highly significant.

For Experiment 2, data for each rat was ‘normalized’ to individual baselines. Log transformations of the data were performed to reduce the influence of variance associated with group means. Descriptive statistics of mean, median and standard deviation were computed for each of the four treatments groups (Groups I-IV) at each of the three time-points (A, B, C). Mixed models methods using repeated measures were used to estimate the rate of change over time (slope), and to test these slopes among the four groups. *P*-values between 0.01 and 0.05 were interpreted as significant whereas *P*-values <0.01 were considered highly significant.

## Results

**Figure 1:**
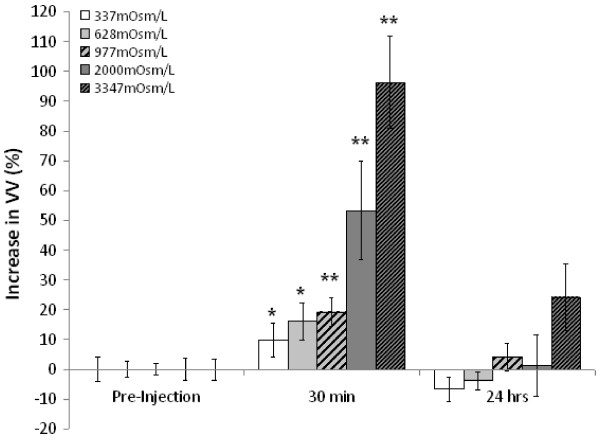
**Experiment 1: VV was measured 30 minutes and 24 hours following acute intraventricular injection of 10kD dextran solution yielding a range of CSF osmolarities spanning an order of magnitude.** Results are expressed as a percentage relative to normalized VVs measured prior to ventricular injection. VVs were significantly increased for all groups at 30 minutes post-injection (*P* < 0.037), with highly significant increases for Groups III (977 mOsm/L), IV (2000 mOsm/L) and V (3347 mOsm/L) (*P* < 0.007). These increases in VV were completely resolved by 24 hours post-injection. Significant differences in VV between groups were not observed either pre-injection or 24 hours post-injection. At 30 minutes post-injection, there is a positive correlation (R^2^ = 0.8349) between increased CSF osmolarity and VV. (* *P* < 0.05, ** *P* < 0.01).

For Experiment 1 (Table 1 and Figure 1), there was a positive correlation between VV and the osmolarity of dextran solution at 30 minutes post-injection (r^2^ = 0.8349, *P* < 0.001), which was not present pre-injection or at 24 hours post-injection. Within group comparisons of pre-injection VVs relative to VVs at 30 min and 24 hours post-injection revealed that VVs were significantly increased for all groups at 30 minutes post-injection (*P* < 0.037), with highly significant increases for Groups III, IV, and V (*P* < 0.007); however, these increases in VV had completely resolved by 24 hours post-injection. Between group comparisons of VVs at each time-point revealed that VV was significantly increased in Group V (*P* < 0.012) at 30 min post-injection relative to Groups I – IV (*P* < 0.017), with a trend toward increased VVs in Group IV (*P* <0.061). Significant differences in VV were not observed between groups either pre-injection or at 24 hours post-injection. Moreover, there were no significant differences between pre-injection VVs and VVs at 24 hours post-injection, reflecting that fact that VVs returned to their original pre-injection volumes by 24 hours post-injection.

**Table 1:** Non-normalized mean ventricle volumes for Experiments 1 and 2

**Experiment 1**	**Pre-Injection ± SD (μm**^**3**^**)**	**30 Minutes Post-Injection ± SD (μm**^**3**^**)**	**24 hours Post-Injection ± SD (μm**^**3**^**)**	
**337mOsm/L (n=10)**	16.69 ± 2.2	18.3 ± 3.04	15.75 ± 2.12	
**628mOsm/L (n=10)**	16.42 ± 1.37	19.06 ± 3.28	15.79 ± 1.56	
**977mOsm/L (n=9)**	16.29 ± 1.07	19.42 ± 2.51	16.97 ± 2.38	
**2000mOsm/L (n=8)**	16.17 ± 1.88	24.79 ± 8.51	16.4 ± 5.26	
**3347mOsm/L (n=8)**	15.7 ± 1.78	30.81 ± 7.69	19.5 ± 5.63	
**Experiment 2**	**Pre-Surgery** (**μm**^**3**^)	**Pump On (μm**^**3**^**) Interval A**	**Pump Off/On (μm**^**3**^**) Interval B**	**Pump On/Off (μm**^**3**^**) Interval C**
**On-Off-On (337mOsm/L, n=9) ± SD**	19.16 ± 3.78	25.98 ± 8.96	26.67 ± 10.35	28.63 ± 11.69
**On-Off-Off (337mOsm/L, n=10) ± SD**	16.63 ± 1.85	19.02 ± 2.55	21.7 ± 6.68	20.2 ± 3.38
**On-On-On (337mOsm/L, n=10) ± SD**	14.47 ± 2.36	24.09 ± 13.06	27.64 ± 12.98	32.95 ± 19.33
**On-On-On (307mOsm/L, n=10) ± SD**	17.23 ± 1.96	19.75 ± 4.22	20.26 ± 4.51	21.56 ± 6.02

**Figure 2:**
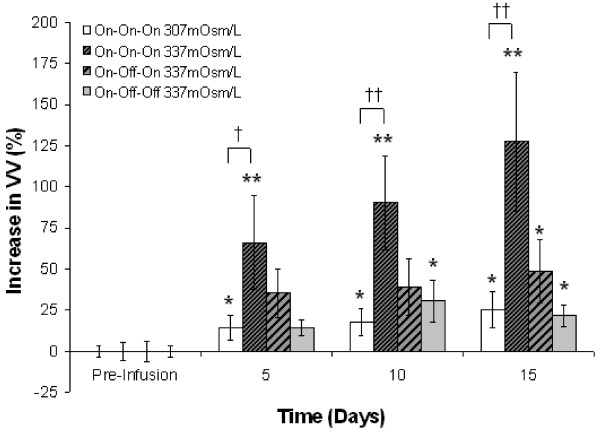
**Experiment 2: VV was measured at 5 day intervals during continuous intraventricular infusion of 10kD dextran yielding CSF osmolarities of 307 (isoosmolar) or 337 mOsm/L (hyperosmolar).** Results are expressed as a percentage relative to normalized VVs measured prior to initiating ventricular infusion. VV in Group I (On-On-On 307 mOsm/L) increased incrementally at each time-point over the course of continuous iso-osmotic dextran infusion. VV in Group II (On-On-On 337 mOsm/L) also increased at each time-point over the course of continuous hyper-osmotic dextran infusion; however, these increases were larger and highly significant. VV in Group III (On-Off-On 337 mOsm/L), trended toward increase during interval A, slowed during interval B when infusion was suspended, and resumed increasing and reached significance during interval C when infusion was restored. VV in Group IV (On-Off-Off 337 mOsm/L), trended toward increase during interval A. The rate of increase slowed during intervals B and C when infusion was suspended but the increase in VV reached significance at both time-points.

**Figure 3:**
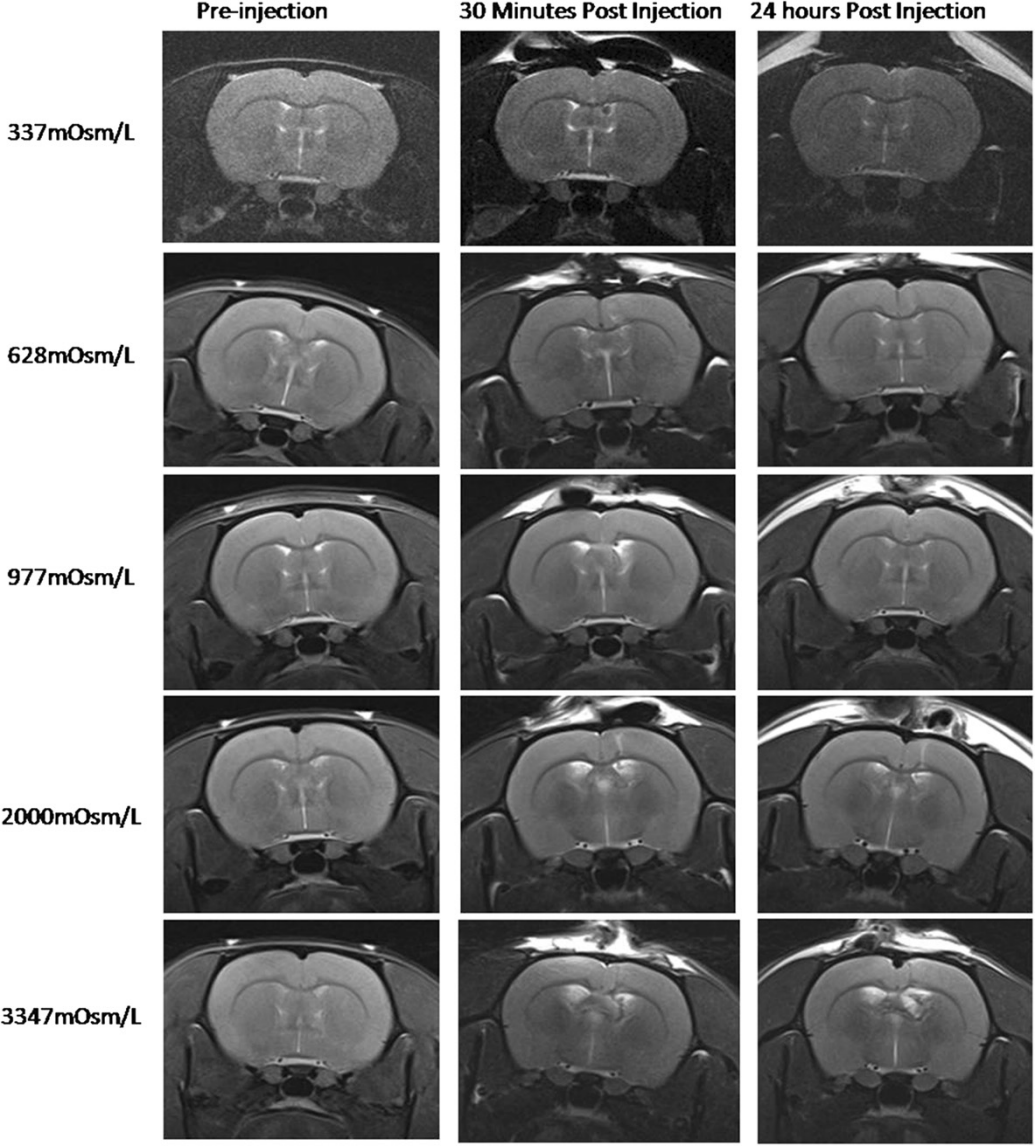
**Coronal T2-weighted MR images obtained from representative animals from Group I (337 mOsm/L), Group II (628 mOsm/L), Group III (977 mOsm/L), Group IV (2000 mOsm/L) and Group V (3347 mOsm/L) in Experiment 1.** MR images were acquired pre-infusion (column 1), at the end of 30 mins (column 2) and at 24 hours (column 3) following injection.

**Figure 4:**
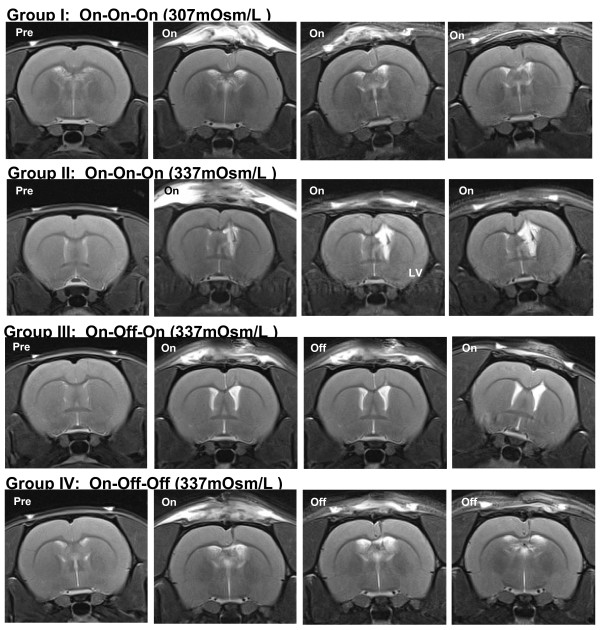
**Coronal T2-weighted MR images obtained from representative animals from Groups I, II, III and IV in Experiment 2.** MR images were acquired pre-infusion (column 1), and at the end of intervals A (column 2, days 1–5), B (column 3, days 6–10) and C (column 4, days 11–15). LV: lateral ventricle.

For Experiment 2 (Table 1 and Figure 2), the infused dextran solution was iso-osmotic (307 mOsm/L) for Group I and mildly hyper-osmotic (337 mOsm/L) for Groups II, III, and IV. VV was assayed before and at 5 day intervals after initiating dextran infusion, immediately prior to manipulations of the infusion status (On/Off). Within group comparisons revealed that, relative to preinfusion values, VV in Group I (On-On-On, n = 10) increased incrementally at each time-point over the course of continuous iso-osmotic dextran infusion (A: *P* < 0.044, B: *P* < 0.023, C: *P* < 0.015). VV in Group II (On-On-On, n = 10) also increased at each time-point over the course of continuous hyper-osmotic dextran infusion; however, these increases were larger and highly significant (A: p < 0.007, B: *P* < 0.001, C: *P* < 0.001). VV in Group III (On-Off-On, n = 9), trended toward increase during interval A (*P* < 0.069); however, the rate of increase slowed significantly during interval B when infusion was suspended (*P* < 0.061) and resumed increasing at its original rate and reached significance during interval C when infusion was restored (*P* < 0.028). VV in Group IV (On-Off-Off, n = 10), trended toward increase during interval A (*P* < 0.081). The rate of increase slowed during intervals B and C when infusion was suspended but the increase in VV reached significance at both time-points (B: *P* < 0.045, C: *P* < 0.009). Between group comparisons of VVs at each time-point revealed that, relative to Group I, the increase in VV in Group II was significant during interval A (*P* < 0.047), and highly significant during intervals B (*P* < 0.001) and C (*P* < 0.001). The increase in VV in Group II, relative to Group IV, was also significant during interval B (*P* < 0.017) and highly significant during interval C (*P* < 0.001). MRI images (Figure 3) revealed no evidence of obstruction or occlusion of the cerebral aqueducts or other putative CSF circulation pathways. Representative T2 weighted MR images of different groups depicting changes in VV are presented in Figure 4.

Behavioral effects related to these manipulations of CSF osmolarity and VV were not systematically investigated; however, with the exception of transient anesthesia effects, the rats appeared alert and attentive and maintained their weight and hydration status for the duration of the study.

## Discussion

The objective of this study was to elucidate the kinetics associated with manipulating CSF osmolarity on VV in the normal rat brain. Our results confirm previous reports suggesting that increasing CSF osmolarity is sufficient to increase VV and include novel observations regarding the influence of concentration and time in this context. Transient increases of CSF osmolarity over a range spanning an order of magnitude are sufficient to cause acute and proportional increases in VV which are completely reversed by 24 hours post-injection. These acute increases in VV are observed primarily within the injected hemisphere at 30 minutes post-injection but encompass the entire rostral-caudal extent of the brain. Sustained increases of CSF osmolarity at the lower end of this range result in more gradual increases in VV that evolve bilaterally over the course of five days of continuous dextran infusion and are much slower to reverse when dextran infusion is suspended. This apparent hysteresis may reflect a persistent saturation of clearance mechanisms which normally regulate CSF osmolarity and/or ventricular volume. Collectively, our results suggest that the induction and withdrawal kinetics of increased VV are directly affected by both the magnitude and duration of increases in CSF osmolarity.

Changes in VV produced by manipulating CSF osmolarity may reflect the relatively free movement of water within brain tissues made possible by the presence of aquaporins 1, 4, and 9 [34-36]. These aquaporins allow the water component of CSF and plasma to move freely across the BBB in response to osmotic gradients associated with the presence of relatively impermeable and strictly regulated solutes. Transport of these solutes might, therefore, be a means by which CSF osmolarity and the volume of CSF is normally regulated within the ventricles. Recent reports suggest that water may cycle continuously between CSF and plasma in response to osmotic gradients and changes in hydrostatic pressure, as blood passes from arterial to venous microvessels [37-39]. Consistent with this hypothesis, increases in venous pressure have recently been shown to be sufficient to cause an accumulation of CSF within the ventricles [37].

The rapid induction and withdrawal kinetics of VV increases associated with transient increases in CSF osmolarity, suggest that 10 kD dextran is rapidly and efficiently cleared from CSF under these conditions. It remains to be determined whether the associated ipsilateral increase in VV observed at 30 minutes post-injection eventually becomes bilateral or whether the rate of clearance is sufficient to prevent this. By contrast, the much more gradual induction and withdrawal kinetics of VV increases induced by modest increases in CSF osmolarity sustained over several days were unexpected. These may reflect a gradual saturation of endogenous transport mechanisms which normally participate in clearing such solutes from CSF, combined with a long post-saturation refractory period. An analogous influx of solutes into CSF resulting from brain tumors, cerebral trauma, arterio-venous malformations, neurodegenerative diseases and other brain pathologies might, therefore, similarly saturate these putative transport mechanisms and give rise to hydrocephalus displaying similar kinetics. Such solutes could include ions, amino acids, neurotransmitters, proteins and peptides crossing the compromised BBB and other cellular debris associated with apoptotic and/or necrotic cell death.

In conclusion, our results suggest that brain pathologies that compromise BBB integrity or which otherwise promote the pathological release of cellular constituents into interstitial fluid and/or CSF may be sufficient to induce hydrocephalus by increasing CSF osmolarity. Moreover, a sustained influx of these constituents at relatively low concentrations and the associated increase in CSF osmolarity may be more pathogenic than an acute influx at higher concentrations. Therapies which prevent or mitigate such increases in CSF osmolarity might therefore prove useful in treating hydrocephalus. Assays of CSF content that affects osmolarity might also prove useful for distinguishing among types of hydrocephalus that may be responsive to such a therapeutic approach.

## Competing interests

The authors have no competing interests.

## Authors’ contributions

SK designed the study, was principle investigator and primary author. J L performed the experiments, obtained the MRI scan data, assisted in designing the study and writing the article. LS performed statistical analysis. KAJ served as a consultant for data analysis and interpretation and provided extensive editorial support in drafting the manuscript. All authors have read and approved the final version of the manuscript.
